# Pentraxin 3 (PTX-3) Levels in Bronchoalveolar Lavage Fluid as a Lung Cancer Biomarker

**DOI:** 10.1155/2020/4652483

**Published:** 2020-06-10

**Authors:** Tinghua Hu, LiBang Qiao, Hong Li, Hui Ren, Qian Ning, Hong Zhou, Xue Chen, Zhongmin Sun, Liangrong Shen

**Affiliations:** ^1^Department of Respiratory and Critical Care Medicine, First Affiliated Hospital of Xi'an Jiaotong University, Xi'an, Shaanxi, 710061, China; ^2^Central Sterile Supply Department, First Affiliated Hospital of Xi'an Jiaotong University, Xi'an, Shaanxi, 710061, China

## Abstract

In this study, we investigated the utility of pentraxin 3 (PTX-3) in bronchoalveolar lavage fluid (BALF) as lung cancer (LCa) diagnostic. A total of 89 LCa patients and 84 non-LCa patients who received bronchoscopy in the First Affiliated Hospital of Xi'an Jiaotong University from December 2014 to February 2015 were enrolled. LCa was subdivided according to pathological type (scale, gland, and small cell lung cancer). BALF samples were obtained during bronchoscopy and PTX-3 levels assayed by ELISA. *t*-tests, Mann-Whitney, and Kruskal-Wallis tests were performed for the comparison of PTX-3 levels between the different groups. Correlation analysis and receiver operating characteristic (ROC) analysis were used to analyze clinical data. The levels of PTX-3 increased in the LCa groups. PTX-3 levels were higher in the small cell lung cancer (SCLC) compared to non-small-cell lung cancer (NSCLC) groups. In LCa patients, obstructive pneumonia could upregulate the expression of PTX-3 in BALF. The area under the ROC curve of PTX-3 in BALF during LCa diagnosis, SCLC, and LCa with obstructive pneumonia was 0.949 (*p* ≤ 0.001), 0.672 (*p* < 0.05), and 0.838 (*p* < 0.01), respectively. In conclusion, PTX-3 in BALF has a potential value as an LCa biomarker, particularly in cases of SCLC and LCa with obstructive pneumonia.

## 1. Introduction

Lung cancer (LCa) is the leading cause of cancer-related death globally [1]. Due to poor LCa detection methods, only ~10-20% of patients with LCa can be treated at the time of diagnosis [2] leading to 5-year survival rates of ~14% [3]. Current LCa markers include carcinoembryonic antigen, squamous cell carcinoma antigen, neuron-specific enolase (NSE), cancer antigen 125, and cytokeratin 19 fragment, but all lack the sensitivity and specificity to act as early LCa diagnostics [4].

Pentraxin 3 (PTX-3) is a long pentraxin [5, 6] produced locally at sites of inflammation by an array of immune cells in response to inflammatory signals including IL-1, TNF*α*, toll-like receptor, and LPS [7, 8]. The induction of PTX-3 is fast and short-lived, peaking 4–6 hours poststimulation [9], but can be trapped by neutrophils to enhance its capability to eliminate pathogens and prevent necrosis and apoptosis [10]. PTX-3 also participates in an array of biological effects, including immune defenses, inflammation, and apoptosis [11]. In recent years, it has been reported that PTX-3 played an important role in respiratory diseases, such as cigarette-induced pulmonary emphysema [12], lung infections [13, 14], severe acute respiratory syndrome [15], and pulmonary arterial hypertension [16].

Previous studies on the expression of PTX-3 have focused on serum and lung tissue [17, 18]. However, studies on the expression of PTX-3 in alveolar lavage are sparse. In this study, through the detection of PTX-3 expression in the bronchoalveolar lavage fluid of patients suffering from LCa, we explored its role in the early diagnosis of LCa.

## 2. Methods

### 2.1. Patient Cohort

Patients receiving bronchoscopy at our institute from December 2014 to February 2015 were selected. All of the preoperative preparations were improved, including informed consent for bronchoscopy and anesthesia.

Inclusion criteria were as follows: (1) aged ≥18 years; (2) undergoing bronchoscopy; and (3) pathological diagnosis of LCa.

Exclusion criteria are as follows: (1) long-term oral intake of corticosteroids; (2) adrenal; (3) unqualified bronchoalveolar lavage fluid; and (4) patients who do not meet the inclusion criteria.

### 2.2. Bronchoalveolar Lavage Fluid (BALF) Collection

Through electrocardiograph monitoring, blood oxygen saturation, and anesthesia, electronic bronchoscopes (BF260, FF260, BF240, Olympus, Tokyo, Japan) were used to collect the BALFs, with all stages performed according to the conventional bronchoscopy process [19]. The bronchoscope entered the lung segments or subsegments near the lesions (chest CT suggestive of exudation, new infections, and so on). Bronchoalveolar lavage was performed on the collected BALF with recovery rates of 40%-60%. BALF samples were centrifuged (10 min at 3000 rpm); the supernatants were collected and all samples were stored at -80°C.

### 2.3. PTX-3 Detection

PTX-3 levels in BALF were detected by ELISA as per the manufacturer's instructions (R&D Company, USA).

### 2.4. General Information and Laboratory Parameters

General clinical data and laboratory parameters were obtained from clinical records, laboratory assessments, biochemical rooms, and imaging analysis. Times between chest CT scans and bronchoscope examinations did not exceed 1 week. Blood biochemical specimens and bronchoscope examinations lasted no more than 3 days.

### 2.5. Statistical Analysis

SPSS 20.0 statistical software was used for statistical analysis. Quantitative data conformed to a normal distribution and are shown as the mean ± standard error. Quantitative data belonging to abnormal distributions was presented as the median and quartile range. Groups were compared via *t*-tests, Mann-Whitney tests, and Kruskal-Wallis analysis. Spearman's tests were used for correlation analysis. The diagnostic values of PTX-3 in BALF were analyzed by ROC curves. *p* values < 0.05 were deemed significant differences.

## 3. Results

### 3.1. Patient Data

A total of 173 BALF samples were collected by bronchoscopy. A total of 89 patients had LCa, amongst which 67 were male and 22 were female, with an average age of 60.76 ± 9.63 years. A total of 84 patients were LCa negative, amongst which 50 were male and 33 were female, with an average age of 54.9 ± 15.73 years.  Table 1 shows all patient characteristics.

### 3.2. PTX-3 Expression in BALF in LCa vs. Non-LCa Patients

**Figure 1:**
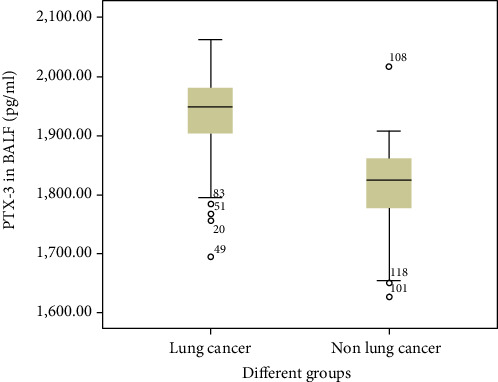
Expression of PTX-3 in BALF is upregulated in lung cancer patients. PTX-3 levels were detected by ELISA. Means between groups with and without LCa were compared.

**Figure 2:**
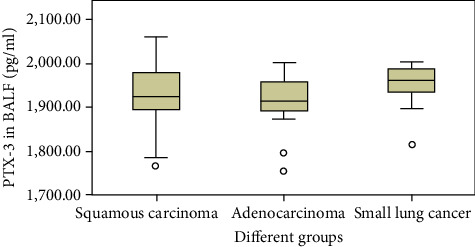
PTX-3 in BALF in the three pathology subgroups.

LCa groups showed higher levels of PTX-3 in BALF (Figure 1 and Table 2). Of the patients, 30 had scale cancer, 24 had gland cancer, 21 had SCLC, and 14 had no pathological diagnosis (Table 3). As shown in Figure 2, PTX-3 expression significantly differed across the groups (*p* < 0.05), particularly in cases of gland lung cancer vs. SLCC (*p* < 0.01). No significant differences were observed between scale cancer and gland LCa or scale LCa and SCLC (*p* > 0.05).

### 3.3. PTX-3 Expression in BALF with or without Obstructive Pneumonia

**Figure 3:**
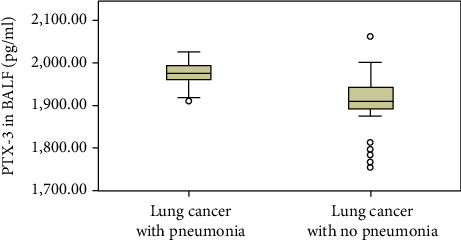
Expression of PTX-3 in BALF in lung cancer patients with or without obstructive pneumonia.

Obstructive pneumonia is the main X-ray feature of LCa that was judged according to the following criteria [20]: patchy infiltration in chest CT and at least 2 of the following 4 items: temperature is more than 38°C; blood leukocyte counts > 10 × 10^9^/L or <4 × 10^9^/L; purulent secretions in the respiratory tract; and positive bacterial cultures of respiratory secretions. The LCa group was subdivided into two groups: namely, those with and without obstructive pneumonia. Group comparisons showed significant differences (*p* < 0.01) in PTX-3 in BALF groups (Table 4, Figure 3).

### 3.4. Correlation between PTX-3 in BALF and Specific Clinical Indicators

Spearman correlation analysis was performed to compare PTX-3 levels in BALF and serum CEA, NSE, and CYFRA 21-1 and the smoking index, respectively. The results showed that CEA, NSE, and CYFRA 21-1 positively correlated with PTX-3 levels in BALF with no statistical significance, whilst for the smoking index, a negative correlation was observed (Table 5).

### 3.5. Diagnostic Values of PTX-3 in BALF and Serum CEA, NSE, and CYFRA 21-1 for LCa

**Figure 4:**
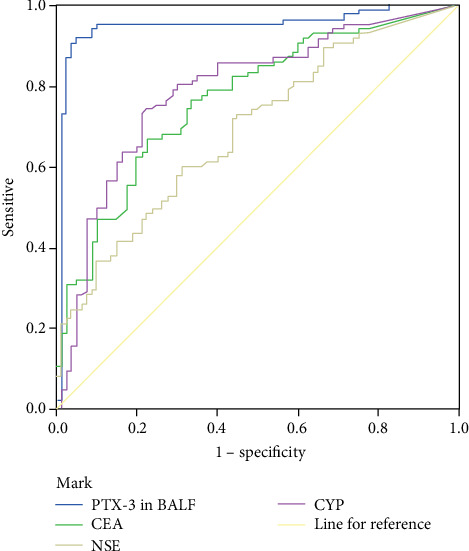
ROC curves of the diagnostic value of PTX-3 in BALF and serum CEA, NSE, and CYFRA 21-1 for LCa.

We applied ROC curve analysis to assess the diagnostic value of PTX-3 in BALF. Serum CEA, NSE, and CYFRA 21-1 for LCa are shown in Figure 4 and Table 6. The PTX-3 in BALF showed a diagnostic advantage in lung cancer diagnosed with a larger area under the curve (0.949). When taking the cutoff point of 1889.8286 pg/mL for PTX-3 level, the sensitivity for LCa diagnosis was 88.8%, and the specificity was 96.4%.

### 3.6. Diagnostic Value of PTX-3 in BALF for SCLC

**Figure 5:**
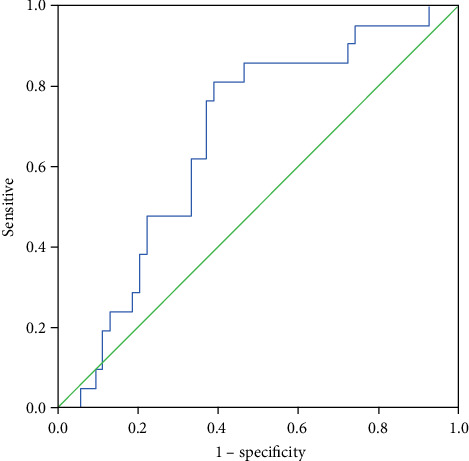
ROC curves of PTX-3 in BALF for the diagnosis of small cell lung cancer.

The AUC of the ROC curves (Figure 5) indicated that the diagnostic value of PTX-3 for BALF in SCLC was 0.672 (95% CI; 0.543, 0.801, *p* < 0.05). When taking the cutoff point of 1933.0837 pg/mL for PTX-3 level, the sensitivity for the diagnosis of SCLC was 81.00%, and the specificity was 61.10%. This suggested that the PTX-3 levels in BALF can distinguish SCLC and NSCLC.

### 3.7. Diagnostic Value of PTX-3 in BALF for LCa Patients with Obstructive Pneumonia

**Figure 6:**
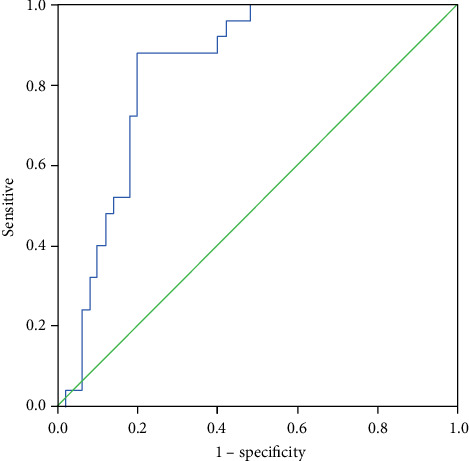
ROC curves of PTX-3 in BALF for the diagnosis of lung cancer with obstructive pneumonia.

Figure 6 shows that the AUC of ROC for PTX-3 in BALF for LCa with obstructive pneumonia was 0.838 (95% CI; 0.747, 0.928; *p* < 0.05). At the cutoff point of 1952.3828 pg/mL for PTX-3 levels, the sensitivity for the diagnosis of lung cancer with obstructive pneumonia was 88.00% and the specificity was 80.00%. Thus, PTX-3 levels in BALF had a diagnostic value for LCa with obstructive pneumonia.

## 4. Discussion

Bronchoalveolar lavage fluid (BALF), also termed liquid biopsy, has great importance and significance in early diagnosis, differential diagnosis, judgment of curative effects, and the prognostic evaluation of respiratory disease.

Known as one type of soluble pattern recognition receptor, PTX-3 plays a key role in immune defenses. It can be produced from various cell types including immunocytes and histiocytes and participants in (TLR) responses, inflammation, and physical and chemical injuries [21]. Compared to common pentraxins such as CRP, PTX-3 differs in terms of source, distribution, identifiable ligands, and induction signals. In addition, PTX-3 is long lasting, has a stable genetic structure, and is not easily influenced by gender, body weight, and liver or kidney function. PTX-3 thus shows significant advantages over CRP for local inflammatory reactions [22].

PTX-3 is an inflammatory indicator often considered as a modulator of tumor-associated inflammation [23], with important roles in an array of cancers [24–26]. The cutoff value of PTX-3 in the serum is 8.03 ng/mL and can be used as a diagnostic for LCa, with a sensitivity of 72.8% and a specificity of 77.3% [4]. We investigated the levels of PTX-3 levels in lung tissues and serum from the lower respiratory tract and found that PTX-3 levels in the BALF of patients with LCa were significantly higher than those of patients without LCa.

PTX-3 is a biomarker for various inflammatory conditions including febrile neutropenia [27]. We found that the PTX-3 levels in the BALF of patients suffering from LCa with obstructive pneumonia were much higher. Upon comparison of scale lung cancer, gland lung cancer, and SCLC, PTX-3 levels in the BALF of patients suffering from small cell lung cancer were drastically increased (cutoff value: 1933.0837 pg/mL; sensitivity for diagnosis: 81.00%; specificity: 61.10%). This prompted us to conclude that PTX-3 levels in BALF have diagnostic values for these LCa subtypes, which was consistent with other studies [28].

A range of tumor markers has been adopted for LCa in the clinic including CA19.9 CA125, TAG-72.3, CA15.3, CEA, SCC, SNSE, and CYFRA 21-1. However, due to their lack of sensitivity and specificity, particularly in patients with renal failure and hepatic diseases, false positives and negative are frequently encountered, limiting their applicability for mass screening or auxiliary diagnosis. PTX-3 levels in BALF are largely unaffected by gender, age, basic vital signs, peripheral blood leukocyte count, or neutrophil counts. Compared to classical serum LCa biomarkers, PTX-3 in BALF was superior with high diagnostic sensitivity and specificity.

In conclusion, the expression of PTX-3 in BALF is a clinical indicator for the diagnosis of LCa prior to obtaining pathological data.

## Figures and Tables

**Table 1:** Clinical characteristics of the patients.

Item	Patients with lung cancer	Patients without lung cancer	*p* value
Number (*N*)	89	84	—
Sexuality (male/female)	67/22	50/33	—
Age (year)	60.76 ± 9.63	54.9 ± 15.73	*p* > 0.05
*T* (°C)	36.75 (36.50-37.40)	36.87 (36.50-37.05)	*p* > 0.05
Pause (per/min)	82 (76-87.5)	80 (78-88)	*p* > 0.05
Respiration (per/min)	20 (19-21)	20 (20-20)	*p* > 0.05
SBP (mmHg)	120 (110-132)	120 (110-130)	*p* > 0.05
DBP (mmHg)	78 (70-80)	73 (70-80)	*p* > 0.05
WBC count (×10^9^/L)	6.75 (5.79-8.17)	7.15 (4.76-8.76)	*p* > 0.05
Neutrophil count (×10^9^/L)	4.74 (3.73-6.03)	5.04 (3.46-6.78)	*p* > 0.05
Neutrophil percentage (%)	72.36 (64.7-78.6)	73.11 (67.54-80.20)	*p* > 0.05
Monocyte count (×10^9^/L)	0.42 (0.30-0.55)	0.35 (0.24-0.52)	*p* > 0.05
Monocyte percentage (%)	5.75 (4.47-7.20)	5.10 (4.20-6.60)	*p* > 0.05
Pulmonary complications	—	—	—
COPD (*N*)	9	8	—
Bronchiectasis (*N*)	2	2	—
Interstitial disease (*N*)	3	0	—
Pleural effusion (*N*)	22	12	—

**Table 2:** PTX-3 level of patients with lung cancer and without lung cancer.

	Patients with lung cancer	Patients without lung cancer	*p* value
PTX-3 level (pg/mL)	1947.9774 (1904.0944-1979.7761)	1825.0369 (1776.8666-1859.9526)	≤0.000∗∗

∗∗*p* < 0.01.

**Table 3:** General information of the pathology subgroups of the lung cancer group.

Items	Scale cancer	Gland cancer	Small cell lung cancer	Undiagnosed	*p* value
Number (*N*)	30	24	21	14	—
Sexuality (male/female)	(29/1)	(14/10)	(16/5)	(8/6)	—
Age (year)	63 (59-71)	58 (53-62.5)	62 (53-68)	62 (53-67)	*p* > 0.05
*T* (°C)	36.7 (36.5-37.4)	36.7 (36.45-38)	36.8 (36.5-37.1)	36.8 (36.6-37)	*p* > 0.05
Pause (per/min)	82 (76-86)	84 (76-90)	81 (74-84)	81 (78-84)	*p* > 0.05
Respiration (per/min)	20 (19-20)	20 (19.5-21.5)	20 (18-20)	20 (19-21)	*p* > 0.05
SBP (mmHg)	125 (110-132)	126 (118-137)	114 (110-130)	111 (110-130)	*p* > 0.05
DBP (mmHg)	78 (70-80)	78 (72-80)	80 (70-80)	70 (70-80)	*p* > 0.05
WBC count (×10^9^/L)	6.31 (6.02-7.52)	7.08 (6.41-8.34)	6.16 (5.36-7.6)	7.17 (5.18-10.77)	*p* > 0.05
Neutrophil count (×10^9^/L)	4.64 (3.92-5.76)	4.96 (4.06-6.40)	4.61 (3.42-5.93)	5.20 (3.7-8.88)	*p* > 0.05
Neutrophil percentage (%)	76.94 (66.5-81.84)	74.2 (61.2-79.1)	70.97 (65.05-74.2)	70.2 (65.5-75.51)	*p* > 0.05
Monocyte count (×10^9^/L)	0.41 (0.31-0.58)	0.42 (0.29-0.56)	0.45 (0.34-0.49)	0.32 (0.26-0.52)	*p* > 0.05
Monocyte percentage (%)	6.00 (4.7-7.2)	0.42 (0.29-0.55)	0.45 (0.34-0.49)	0.32 (0.26-0.52)	*p* > 0.05

**Table 4:** Comparison between groups with or without obstructive pneumonia.

Group	Median (interquartile range)	*p* value
Patients associated with obstructive pneumonia	1977.8666 (31.1316)	≤0.000∗∗
Patients without obstructive pneumonia	1912.0813 (55.4708)	

∗*p* < 0.05, ∗∗*p* < 0.01.

**Table 5:** Univariate analysis for the correlation between PTX-3 level in BALF and some certain clinical indicators.

Items	The Spearman correlation coefficient	
	*r* value	*p* value
WBC count (×10^9^/L)	0.094	0.385
Neutrophil count (×10^9^/L)	0.170	0.113
Monocyte count (×10^9^/L)	0.003	0.976
CRP (mg/L)	0.198	0.073
ESR (mm/L)	0.230	0.035∗
CEA (ng/mL)	0.003	0.975
NSE (ng/mL)	0.016	0.884
CYFRA 21-1 (ng/mL)	0.170	0.120
The smoking index	-0.289	0.006∗∗

∗*p* < 0.05, ∗∗*p* < 0.01.

**Table 6:** The ROC curve analysis of the diagnostic value of PTX-3 in BALF and serum CEA, NSE, and CYFRA 21-1 for lung cancer.

The biomarkers	Area under the curve (AUC)	95% confidence interval		*p* value
		Lower limit	Upper limit	
The PTX-3 level in BALF	0.949	0.909	0.989	<0.001
CEA in serum	0.767	0.696	0.839	<0.001
NSE in serum	0.689	0.609	0.768	<0.001
CYFRA 21-1 in serum	0.790	0.719	0.861	<0.001

## Data Availability

The data used to support the findings of this study are included within the article.
